# BioCreAtIvE Task1A: entity identification with a stochastic tagger

**DOI:** 10.1186/1471-2105-6-S1-S4

**Published:** 2005-05-24

**Authors:** Shuhei Kinoshita, K Bretonnel Cohen, Philip V Ogren, Lawrence Hunter

**Affiliations:** 1 Center for Computational Pharmacology, University of Colorado School of Medicine, Denver, Colorado; 2 Fujitsu Ltd., BioChemical Information Project, 1-9-3 Nakase Mihama-ku Chiba, JAPAN; 3 Dept. of Computer Science, University of Colorado at Boulder, Boulder, Colorado

## Abstract

**Background:**

Our approach to Task 1A was inspired by Tanabe and Wilbur's ABGene system [1,2]. Like Tanabe and Wilbur, we approached the problem as one of part-of-speech tagging, adding a GENE tag to the standard tag set. Where their system uses the Brill tagger, we used TnT, the *Trigrams 'n' Tags *HMM-based part-of-speech tagger [3]. Based on careful error analysis, we implemented a set of post-processing rules to correct both false positives and false negatives. We participated in both the *open *and the *closed *divisions; for the open division, we made use of data from NCBI.

**Results:**

Our base system without post-processing achieved a precision and recall of 68.0% and 77.2%, respectively, giving an F-measure of 72.3%. The full system with post-processing achieved a precision and recall of 80.3% and 80.5% giving an F-measure of 80.4%. We achieved a slight improvement (F-measure = 80.9%) by employing a dictionary-based post-processing step for the open division. We placed third in both the open and the closed division.

**Conclusion:**

Our results show that a part-of-speech tagger can be augmented with post-processing rules resulting in an entity identification system that competes well with other approaches.

## Background

This paper describes the methods we used to accomplish entity identification (also known as named entity recognition) in the molecular biology domain. Entity identification in this domain has been a subject of interest since Fukuda et al.'s seminal paper on the PROPER/KEX system [4]. The subject is of interest to biologists because it is a necessary first step in many kinds of applications that are of interest to them, including information extraction, information retrieval, and bibliometrics. It is of interest to linguists and computer scientists because it seems to be more difficult than entity identification in "general English" domains [1]. In this paper, we show that a stochastic POS tagger performs well as an entity identification system in the molecular biology domain. Like Tanabe and Wilbur [1,2], we approached the molecular biology entity identification problem as a part-of-speech (POS) tagging task, adding to the standard POS tag set one or more *gene *tags for genes and gene products. Our system replaces the Brill tagger with an HMM-based part-of-speech tagger. Our experience suggests that the Brill tagger is susceptible to specific kinds of performance problems that we hoped to avoid. However, we did not rigorously compare the performance of the two taggers. The main difference between the two systems is our focus on tailoring the post-processing steps for the BioCreAtIvE task. Specifically, we found that understanding the BioCreAtIvE annotation policies for building the corpora and performing error analysis allowed us to create post-processing rules that were effective in increasing performance.

The goal of BioCreAtIvE Task1A is to assess the ability of an automated system to identify mentions of genes in text from biomedical literature. The corpus used for Task1A consists of sentences drawn from Medline abstracts and is divided into three sets: training, devtest, and official test. Table 1 shows the number of sentences and entities for the three subsets. Also shown is the distribution of the lengths (in words) of the gene mentions. Task1A has two divisions: *open *and *closed*. The open division permits systems to use external data resources such as online dictionaries or databases while the closed division does not.

## Results

### Overall

**Figure 1:**
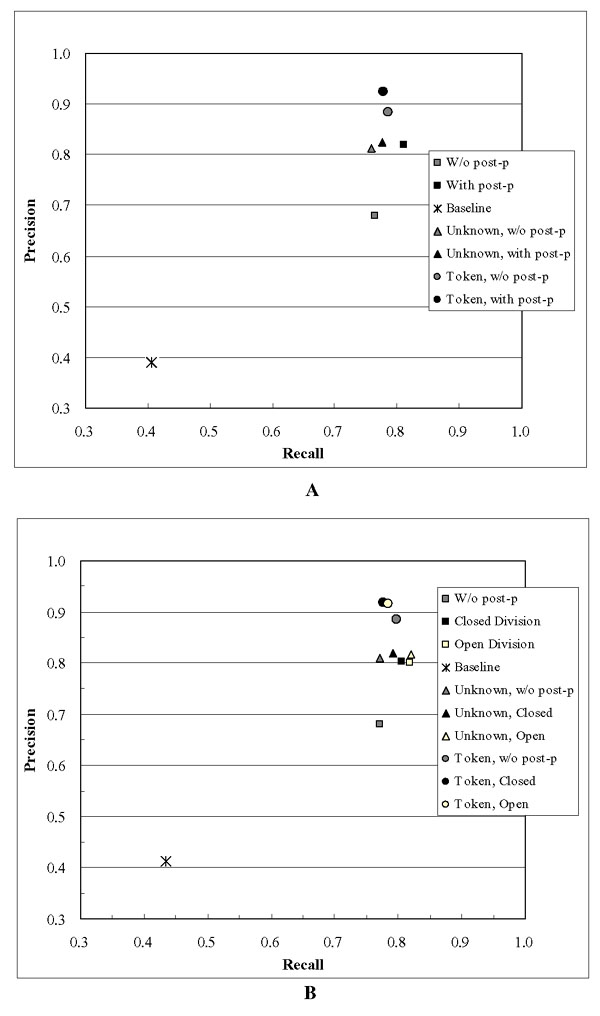
**Precision and Recall. **Figure 1A shows the precision and recall for the cross validation data. Figure 1B shows the precision and recall for the official test data. The expression "w/o post-p" is used as "without post-processing".

We did five rounds of cross-validation, training on four subsets of the data and testing on a fifth using a combined corpus consisting of the training and devtest data. We evaluated our results using the scoring software provided with the BioCreAtIvE data. The resulting average precision and recall were 68.0 and 76.6 with no post-processing (i.e. just based on the output of the tagger). The resulting average precision and recall with post-processing was 82.0 and 81.1, respectively. The averaged results of the cross-validation runs are shown in Figure 1A. The results for official test are shown in Figure 1B. The term-level score comparison between cross-validation and official scores are shown in Table 2. That both sets of results show the same trends shows that our system did not over-train on the devtest corpus and that it performs consistently.

### Term-level precision and recall

Term-level scores (i.e., for performance on full gene names, analogous to the *strict *metric of Olsson et al. [5]) were obtained using the BioCreAtIvE scoring software. We evaluated performance both with and without post-processing. Without performing post-processing, average precision and recall were 68.0 and 76.6. When post-processing was applied, average precision and recall were 82.0 and 81.1. Post-processing improved both the precision and the recall, having a much larger effect on precision than on recall. This tendency is reasonable because our algorithms focus on repairing or removing gene mentions found by the base system and concentrate less on finding new gene mentions that were mistakenly tagged with POS tags such as NN or NNS. A dictionary-based post-processing is introduced to help increase recall. However, the dictionary-based approach increased our F-measure by only 0.5%.

### Baseline, and normalizing for the difficulty of the task

As a baseline for understanding the difficulty of the task, we measured the performance achieved by simply assigning each word the most frequent tag seen with that word in the training set. This baseline strategy achieved an average precision of 39.0 and an average recall of 40.5. For official test the score achieved precision of 41.3 and recall of 43.4. These results are considerably worse than even our without-post-processing results.

### Per-token precision and recall

We then determined the results on a per-word basis. This is equivalent to Olsson et al.'s *protein name parts *metric [5]. In this analysis a true positive is a single word that is tagged as GENE both in the gold standard and by our system. As would be expected, performance on single words is better than the term-level results, with an average precision of 88.3 and average recall of 78.7 without post-processing, and an average precision of 92.5 and average recall of 77.8 with post-processing. Post-processing yielded a 4% improvement in precision, which was less improvement than was seen for full gene names. Recall actually degraded somewhat. These data are consistent with our findings that many of our post-processing steps correct the boundaries of gene mentions at the term level.

### Per-token performance on unknown words

We use the phrase *unknown word *to describe a word that was not previously seen in the training corpora. We used the per-token precision and recall metrics described above to evaluate the performance of our system on unknown words. The cross-validation average precision was 81.3 and average recall was 75.9 without post-processing. Average precision was 82.3 and average recall was 77.6 with post-processing. Post-processing yielded little improvement in performance for unknown words. In the comparison with overall per-token precision and recall (for both known and unknown words), the precision is 10% and 7% worse for with and without post-processing, respectively.

In order to better characterize the effect of unknown words on the performance of our system, we analyzed false positives that are one word in length. The percentage of false positives that are one word long is 40% and 43% for our system without post-processing and with post-processing, respectively. These ratios are similar to the ratio of one-word gene mentions in the corpus given in Table 1. Table 3 shows the effectiveness of post-processing on one-word false positives with respect to the number of times the words corresponding to the false positives were seen in the training data. This table shows that 12.3% of one-word false positives that correspond to unknown words were corrected while 85.2% of one-word false positives that correspond to a word that had been seen twice or more in the training data were corrected. After post-processing is complete, 93% of the remaining one-word false positives correspond to unknown words or words that have been seen only once. This suggests that the lexicon contained in the training data is very important for being able to successfully apply our post-processing steps. We believe that a larger training set covering a larger lexicon would help improve the performance of our system.

### Effect of term length on performance

**Figure 2:**
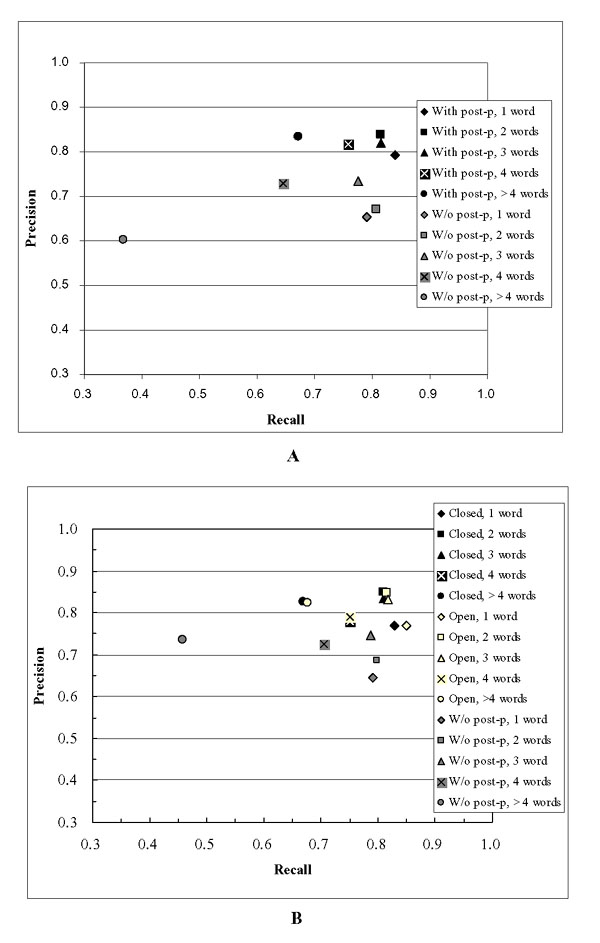
**Effect of term length on performance. **Figure 2A shows the effect of term length for the cross validation data. Figure 2B shows the effect of term length for the official test data.

Figure 2A shows the effect of term length on performance for the cross-validation. Figure 2B shows the effect of term length on performance for the official test. Both figures show the same trends:

1. Recall and precision tend to be better for shorter gene mentions. However precision tends to degrade slightly for gene mentions that are only one word long. As length in words increases there is no drastic drop in performance until length in words reaches five.

2. Post-processing is effective on all gene mentions of any length. However, it seems that improvement in performance is greater for longer gene mentions. This is probably due to lexicon-based post-processing that corrects boundaries.

### Overall effects of post-processing

The main effect of rule-based and lexicon-based post-processing is an increase in precision. In cross-validation for full gene names, average precision increased from 68.0 to 82.0, and average recall increased from 76.6 to 81.1. In the official test, precision increased from 68.0 to 80.3 (closed division) and 80.0 (open division) and recall increased from 77.1 to 80.5 (closed division) and 81.8 (open division). On the level of individual token (including unknown words), post-processing had a much smaller, and not always positive, effect.

The main effect of dictionary-based post-processing is an increase in recall. Recall in the open division is increased by 1.3% from the closed divison recall. Table 4 shows the individual post-processing effects in our cross-validation testing. It shows that removing rule-based post-processing or removing the lexicon-based post-processing from the post-processing steps has nearly the same effect. Removing the abbreviation rules from the post-processing has the least effect, which indicates that it may be less important for the system's overall performance.

## Discussion

The results of this study raised four questions that we believe should be addressed in the near future – two general to the entire effort, and two specific to our system.

First, it would have been useful to have an estimate of the upper bound on accuracy for any entity identification system trained on the BioCreAtIvE corpus, which is a function of how consistent and correct that data is. Assessing the inter-reviewer reliability for this corpus and/or an assessment of the corpus by independent human judges would be very helpful in understanding the difficulty of the task. Our guess is that an F-measure of 80 is probably within seven points of the upper limit.

Another important question that arises from this effort is to determine the effect of training corpus size on performance. This could be achieved by training on successively bigger percentages of the training corpus. If performance flattens off before the entire corpus is used then simply increasing corpus size may not be useful. However, if the performance has not yet flattened off (or worsened), then there is hope that our system can be improved simply by training on more data.

There are two aspects specific to our system that we would like to explore. The first has to do with deciding which POS tagger is used. A head-to-head competition between the TnT tagger, the Brill tagger, and perhaps others would help determine whether or not the choice of tagger is an important decision. We would look at both the raw performance of each tagger as well as the performance of post-processing rules applied to the results of each tagger. TnT would have an unfair advantage since the error analysis was performed on its output. Additional error analysis could be done on the output of the other systems. It may also be useful to combine the outputs of multiple taggers as well.

The second aspect has to do with the use of dictionaries. Our system used a simple algorithm to exploit a single data resource from the NCBI. It would be informative to have head-to-head competitions between multiple data resources (and various combinations of them) as well as compare algorithms for making use of these resources. Our study suggests that this would be helpful for improving recall at least modestly.

## Conclusion

The POS-tagging-based approach that we took from the ABGene system worked reasonably well. Post-processing rules, which included pattern-based rules, rules that used abbreviation recognition heuristics, and lexicon-based rules, worked well to increase both precision and recall. The overall F-measure rose from 72.3 (without post-processing) to 80.4 (closed division) on the official test. Our use of domain-specific dictionaries was less effective, giving an increase of only 0.5 in F-measure to 80.9(open division) compared to the post-processing without dictionaries approach. Our conclusion is that either much more sophisticated algorithms that make use of dictionaries need to be employed, or the dictionaries themselves are not sufficient.

## Methods

### The POS tagger

Past experience with the ABGene system in our lab suggested that the POS-tagging-based approach to entity identification is workable in the molecular biology domain. Previous experiments with the TnT *Trigrams 'n' Tags *POS tagger, using the GENIA corpus for cross-validation, showed good results with no post-processing of the output. The TnT system is a stochastic POS tagger, described in detail in Brants (2000). It uses a second-order Markov model with tags as states and words as outputs. Smoothing is done with linear interpolation of unigrams, bigrams, and trigrams, with λ estimated by deleted interpolation. Unknown words are handled by learning tag probabilities for word endings. As a POS tagger, the system has been tested on two languages, viz. English and German. It is publicly available at . We were impressed by its availability on a variety of platforms, its intuitive interface, and the stability of its distribution, which installed easily and never crashed. For the official test we trained TnT on both the training corpus and devtest corpus and then tested it on the official test set. Performance of this system on the official test data, calculated by the BioCreAtIvE scoring software, was P = 68.0, R = 77.1, and F-measure = 72.3.

### Choosing the tag set

Each token in the task1A corpus is labeled with a POS tag or a gene tag. Because the default tagging seemed overly simplistic, we hypothesized that expanding the gene tag set to incorporate boundary information would improve performance. We tested the following gene tag sets:

#### Tag set 1: BioCreAtIvE default gene tag set

The default gene tag set contains two gene tags: 'NEWGENE' and 'NEWGENE1'. The latter tag is used when two gene mentions are immediately next to each other in the text. Approximately 1.1% of the gene mentions in the training and devtest sets are tagged with the 'NEWGENE1' tag. This scheme has two obvious potential disadvantages: one of the tags is under represented and there is no semantic difference between two tags.

**Example: **the/DT dnHLH/NEWGENE protein/NEWGENE Id1/NEWGENE1 inhibits/VBZ

#### Tag set 2: Detailed boundary information

This tag set contains four gene tags: 'GENE_BEGIN', 'GENE_INSIDE', 'GENE_END', and 'GENE_ONEWORD'. These tags incorporate boundary information for multi-word gene mentions and identify single word gene mentions.

**Example: **androgen/GENE_BEGIN receptor/GENE_END (AR/GENE_ONEWORD)

**Example: **Syn/GENE_BEGIN 5/GENE_INSIDE locus/GENE_END

#### Tag set 3: Simplified boundary information

This tag set is a simplified version of tag set 2. It contains two tags: 'GENE_BEGIN' and 'GENE_INSIDE'. Tokens that were tagged 'GENE_END' are now tagged 'GENE_INSIDE' and tokens that were tagged 'GENE_ONEWORD' are now tagged 'GENE_BEGIN'.

**Example: **androgen/GENE_BEGIN receptor/GENE_INSIDE (AR/GENE_BEGIN)

**Example: **Syn/GENE_BEGIN 5/GENE_INSIDE locus/GENE_INSIDE

#### Tag set 4: Simplest tag set

This tag set is a simplified version of tag set 1. Tokens that were tagged 'NEWGENE' or 'NEWGENE1' are all tagged 'GENE'. Thus, there is only one gene tag in this set: 'GENE'

**Example: **the/DT dnHLH/GENE protein/GENE Id1/GENE inhibits/VBZ

For each tag set we modified the training corpus to comply with the tag set and then trained TnT. We tested the four models on the devtest set. The result of this experiment is shown in Table 5. The differences in performance between the four tag sets are very small. The two tag sets that incorporated boundary information performed the worst. This may be because larger tag sets are sometimes harder to learn because there are fewer examples for each tag. We speculate that tag sets two and three could possibly outperform the others if we had more training data. However, because the simplest tagging scheme performed the best, we used this scheme for all subsequent experiments described below.

## Abbreviations

There are many instances in the corpora in which a full gene name is immediately followed by an appositive parenthesized symbol or abbreviation. In many cases, the tagger would recognize either the full gene name or the symbol/abbreviation, but not both. In order to correct these cases we implemented Schwartz and Hearst's [6] algorithm to recognize abbreviations and their appositive long forms, such as *Insulin-like growth factor 1 (IGF-1)*. In this example, the long form is *Insulin-like growth factor 1 *and the abbreviation is *IGF-1*. We developed a number of rules that we applied to long form/abbreviation pairs found by the Schwartz and Hearst algorithm:

### Rule 1

If the last word of the long form was tagged as a gene, then we changed any non-gene tags in the long form and abbreviation to GENE. For example, if a long form/abbreviation pair contained the tag sequence JJ NN NN GENE (NNP), then we changed the tags to GENE GENE GENE GENE (GENE). Conversely, if the last word of the long form was *not *tagged as GENE, then we changed the tags on the other words (and on the abbreviation) to non-GENE tags.

### Rule 2

If the long form contained any word that was tagged as GENE and that did occur in the training data but never with a GENE tag, or if it contained one of a small set of stop words such as *virus *and *cancer*, then all tags on words in the long form (and the abbreviation) were changed to non-GENE tags.

### Rule 3

If the last word was one of a small list of gene keywords such as *protein *and *factor *derived from the BioCreAtIvE specification, then all tags in the long form (and the abbreviation) were changed to GENE.

### Rule 4

This rule applies only to the open division. If one of the previous rules did not tag the long form and the abbreviation with GENE, then apply the following. If the abbreviation was more than three characters long and was tagged as GENE, then we double-checked it against data from NCBI (see Section *Dictionary-based post-processing *below). If the abbreviation was found in the NCBI data, then we changed all tags on the long form to GENE.

### Rule-based post-processing

We applied a number of simple, pattern-based rules to fix cases where the BioCreAtIvE task definition specified that a different boundary for the gene name than the one returned by the raw tagger output.

• If a word is tagged GENE and is followed by a one of 41 gene keywords such as *gene *and *sequence*, then the tag on the keyword is changed to GENE.

• Y-box/GENE sequence/NN -> Y-box/GENE sequence/GENE

• If a word is tagged GENE and is followed by a number, Roman numeral, or Greek letter, then the number/numeral/letter is tagged GENE.

• ROR/GENE alpha/NN -> ROR/GENE alpha/GENE

• If a word is tagged GENE and it is followed by parenthesized material that is five characters or longer, then the parenthesized material is tagged with GENE.

• hTAF/GENE (II/CD)/SYM -> hTAF/GENE (II/CD)/GENE

• If a word is composed of the characters *A*, *C*, *G*, *T*, *U*, *3'*, or *5' *and is four or more characters long such as 5'-TGACGTCA-3', then its tag is changed to GENE unless the word is followed by *box *or *boxes*. In the latter case the words are tagged NN.

• A word is tagged GENE if it matches one of the following patterns:

• The word starts with the character *p *and is followed by two or more digits, e.g. *p53*, and *p69/71*.

• The word starts with *pp *or *gp *and is followed by two or more digits, e.g. *pp43*, *pp85*, *gp27*, and *gp120 × 41*.

• The word starts with *sup *and is followed by two or more digits, e.g. *sup35 *and *sup45*.

• A term is tagged NN if it contains the word *virus *and matches one of the following patterns:

• The last or second-to-last word of the term contains *virus*, e.g. *type I herpes simplex virus*, *adenovirus*, *reovirus RNAs*, and *rotavirus genome*.

• The term ends with *virus type *followed by a digit or Roman numeral, e.g. *human immunodeficiency virus type 1*, and *T-cell lymphotropic virus type I*.

• If a word contains hyphen and the characters preceding the hyphen are capitalized letters or digits and the material following the hyphen is a gene keyword such as *mutan*t, then it is tagged GENE, e.g. *SH2-mutant*, and *ANP-receptor*. This rule is applied only if the material preceding the hyphen is between 3 and 9 characters long.

• If a words length has less than two characters and contains digits, Greek letters or roman numerals, then it is tagged NN.

• ... alpha/GENE ... -> ... alpha/NN ...

• If the word *mutation *is followed by a word tagged GENE, then the word is tagged NN.

• Syn/GENE mutations/GENE -> Syn/GENE mutations/NN

### Lexicon-based post-processing

Examination of how instances of word types are tagged in the training and devtest corpora's lexicon revealed effective post-processing rules. For the lexicon-based post-processing steps, tag set 2, which has detailed boundary information, is used. We use the phrase *ambiguous type *in this section to refer to word types that are labelled in the corpora as both gene and as non-gene. For example, tokens of the ambiguous type *binding *are tagged as JJ, NN, and GENE_INSIDE. Correctly tagging tokens of ambiguous types is a difficult task.

### Boundary correction

The POS tagger's output sometimes contains boundary errors such as the following:

Output: IgG/GENE_BEGIN binding/GENE_END

Gold Standard: IgG/GENE_ONEWORD binding/NONGENE

Problem: Right boundary is wrong.

Output: regulator/GENE_BEGIN virF/GENE_END

Gold Standard: regulator/NONGENE virF/GENE_ONEWORD

Problem: Left boundary is wrong.

We used a small set of rules to correct boundaries of multi-word gene mentions that had a high likelihood of being incorrectly tagged. We used the training and devtest data to find ambiguous types that have zero or low probability (less than 3%) of having the GENE_BEGIN or GENE_END tag in a multi-word gene name. For all multi-word gene mentions output by the tagger, we check the first word to see if it is on a list of words known not to be tagged GENE_BEGIN. If the word is found on the list, then we change its tag to a non-gene tag and check the next word, iterating until a word not on the list is encountered or until each word in the gene mention has been examined. A similar process is applied to the right edge of the multi-word gene mention using the list of words known not to be tagged as GENE_END. The following lines show the POS counts in the training corpus for the words *binding *and *regulator*.

binding: JJ = 192, NN = 80, GENE_INSIDE = 71

regulator: NN = 22, GENE_END = 3, GENE_INSIDE = 1, GENE_ONEWORD = 1

The ambiguous type *binding *never appears in the training or devtest corpora with the tag GENE_END. This suggests that in unseen sentences that it will not likely appear as the last word of a gene mention. The following examples demonstrate how the boundary correction post-processing step would change two gene mentions that mistakenly include the word *binding*.

Output: IgG/NEWGENE binding/NEWGENE

Boundary Correction: IgG/NEWGNE binding/NN

Output: regulator/NEWGENE virF/NEWGNE

Boundary Correction: regulator/NN viF/NEWGENE

### Single-word false positive correction

We applied a similar process to detect single-word false positives output by the POS tagger using a list of ambiguous types that were observed in the training and devtest data to have a zero or very low probability (less than 5%) of being tagged GENE_ONEWORD. For example, the words *pathway *and *estrogen *are ambiguous types that are seldom if ever tagged as GENE_ONEWORD. The word *pathway *never occurs in the corpora tagged as GENE_ONEWORD while the word *estrogen *is tagged GENE_ONEWORD in one (or 4.5%) of 22 occurrences. The following lines show the POS counts in the training corpus for the words *pathway *and *estrogen*.

pathway: NN = 65, GENE_END = 8, GENE_INSIDE = 1

estrogen: NN = 14, GENE_BEGIN = 5, GENE_INSIDE = 2, GENE_ONEWORD = 1

For tokens of ambiguous types that have a low probability of being tagged GENE_ONEWORD, we change the tag to NN.

### Dictionary-based post-processing in the open division

We employed a dictonary-based post-processing step that uses NCBI LocusLink symbols database for the open division. LocusLink database used for this research has 279,007 symbols that include official symbols or other aliases that are used to refer to a given gene. Table 6 shows the count of symbol per species. Our goal was to improve recall without a decrease in precision. Our approach was to examine previously unseen words that were tagged as nouns and were four or more characters in length. If such a word matched a LocusLink symbol, then we tagged it as GENE. If we did not find it in a LocusLink symbol field, then we queried the NCBI website through Entrez using the nucleotide database and restricting our search to the gene name field. If Entrez returned any items, then we tagged the word as GENE.

## Figures and Tables

**Table 1:** BioCreAtIvE Data Sets

**Set**	**Number of Sentences**	**Number of Entities**	**1 word**	**2 words**	**3 words**	**4 words**	**> 4 words**
**training**	7500	8876	46.1%	25.7%	14.9%	6.6%	6.6%
**devtest**	2500	2975	46.6%	23.9%	15.1%	6.7%	7.7%
**official test**	5000	5949	46.1%	26.7%	14.3%	6.2%	6.7%

This table shows the BioCreAtIvE data including the ratio for the word length, which shows same tendency among sets.

**Table 2:** The term-level score comparison between the cross-validation and official test

	**Label**	**Precision**	**Recall**	**F-measure**
**Cross-validation**	No post-processing	68.0%	76.5%	72.0%
	With post-processing	82.0%	81.1%	81.6%

**Official Test**	No post-processing	68.0%	77.2%	72.3%
	With post-processing	80.3%	80.5%	80.4%
	With post-processing, dictionary	80.1%	81.8%	80.9%

This table shows the term-level scores about the cross-validation data and official test.

**Table 3:** Performance on one-word false positives

**Occurrences**	**without post-p**	**with post-p**	**corrected**	**% corrected**
**0**	818	717	101	12.3%
**1**	162	95	67	41.4%
**2+**	440	65	375	85.2%

**Total**	1420	877	543	38.2%

85% of one-word false positives that correspond to a word, which was seen twice or more times, were corrected with post-processing procedures.

**Table 4:** The effect of the post-processing procedures on overall system performance.

**CV Average**	**All PP**	**No PP**	**No rule**	**No abbreviation**	**No lexicon**
**Precision**	82.0%	68.0%	74.2%	81.0%	73.1%
**Recall**	81.1%	76.6%	80.1%	79.8%	79.8%
**F-mesure**	81.6%	72.0%	77.0%	80.4%	76.3%

**Delta-F**	base	-9.5%	-4.5%	-1.2%	-5.2%

This table shows the effects of each post-processing procedures in comparison with the all post-processing results. For example, No rule column shows the results without rule-based post-processing, that shows 4.5% lower score than All Post-processing in F-measure.

**Table 5:** The results of choosing GENE tag set

	**Tag Set 1**	**Tag Set 2**	**Tag Set 3**	**Tag Set 4**
**Precision**	67.9%	67.0%	66.4%	68.9%
**Recall**	77.2%	78.2%	77.2%	77.3%
**F-measure**	72.2%	72.1%	71.4%	72.9%
**Delta F**	-0.62%	-0.72%	-1.43%	base

This table shows the results of difference from the Tag Set 4, which is our choice.

**Table 6:** Symbol count in LocusLink database

**Species**	**Cow**	**Fly**	**Human**	**Mouse**	**Nematode**	**Rat**	**Yeast**	**Zebrafish**	**Total**
**Count**	2,438	68,805	55,730	69,654	43,805	9,221	5,120	24,234	279,007

This table shows the count of symbol per species.
